# Identification of a Four-lncRNA Prognostic Signature for Colon Cancer Based on Genome Instability

**DOI:** 10.1155/2021/7408893

**Published:** 2021-09-21

**Authors:** Duo Yun, Zhirong Yang

**Affiliations:** ^1^Inner Mongolia Yuanhe Mongolian Traditional Medicine Research Institute, Hohhot City, Inner Mongolia, China; ^2^Department of Surgical, The Fujian Pinghe County People's Hospital, Zhangzhou City, Fujian Province, China

## Abstract

LncRNAs (long noncoding RNAs) are closely associated with genome instability. However, the identification of lncRNAs related to the genome instability and their relationship with the prognosis and clinical signature of cancer remains to be explored. In this paper, we analyzed differential lncRNA expression based on the somatic mutation profiles of colon cancer patients from TCGA database and finally identified 153 lncRNAs that are associated with genome instability in colon cancer. Taking four lncRNAs from these 153, we established a genome-instability-related prognostic signature (GIRlncPSig). By applying the GIRlncPSig, we calculated a risk score for each patient, and using their risk scores, we divided them into low- and high-risk groups. We found that the prognosis between the two risk groups was significantly different, and the results were further verified in different independent patient cohorts. Moreover, we observed that the GIRlncPSig was related to somatic mutation rates in colon cancer, indicating that it may be a potential means of measuring genome instability levels in colon cancer. We also revealed that the GIRlncPSig was correlated with BRAF and DPYD mutation rates and that it may be a potential mutation marker for the BRAF and DPYD gene. In summary, we constructed a genome-instability-related lncRNA prognostic signature (GIRlncPSig), which has a significant effect on prognosis prediction and may allow for the discovery of new colon cancer biomarkers.

## 1. Introduction

As a common malignant tumor [1], approximately 104,610 cases would be diagnosed with colon cancer in 2020 [2]. Despite the current progress in examination and treatment, biomarkers for the early detection and prognosis prediction of colon cancer are still lacking, so its long-term prognosis remains poor [3]. Therefore, to improve the diagnosis and prognosis of colon cancer patients, new biomarkers and prognosis evaluation approaches must be developed. According to past research, genome instability affects cancer development and prognosis [4, 5]. Genome instability refers to alterations in DNA ranging from a single nucleotide variation to changes in the entire chromosome. Based on the level of disruption, genome instability can generally be divided into three categories: nucleotide instability, an increased probability of one or several nucleotide base substitutions, deletions, or insertions; microsatellite instability, the increase or decrease of short nucleotide repeats (microsatellites) caused by defects in mismatch repair genes; and chromosomal instability, changes in chromosome number or structure [6]. In colon cancer, genome instability is closely associated with patient treatment. In 2017, the FDA approved pembrolizumab and nivolumab in 2017 as the second-line treatment for colorectal cancer patients with mismatch repair defects or high levels of microsatellite instability [7].

Long noncoding RNAs (lncRNAs), a common type of noncoding RNA that are over 200 nucleotides in length, play a significant role in cancer [8]. Its abnormal expression or behavior has an immense effect on the occurrence of cancer [9, 10]. For example, the lncRNA HOTAIR can downregulate the miRNA-34a and promote the development of colon cancer [11]. Another study found that the lncRNA FENDRR can inhibit the SOX4 protein and impede colon cancer development [12]. Moreover, the lncRNA CYTOR was found to influence the Wnt/*β*-catenin signaling, thereby promoting colon cancer metastasis [13]. These studies indicate that lncRNAs can influence the development and prognosis of the colon cancer. Additionally, many studies have reported that lncRNAs are associated with genome stability; for example, Cusanelli et al. [14] observed that LncRNA TERRA is important in genome stability and telomere maintenance. Betts et al. [15] revealed that the lncRNAs CUPID1 and CUPID2 affect DNA repair and recombination. Although various studies have found that lncRNAs play an important role in tumor genome stability, their specific mechanisms and clinical relevance remain to be further explored.

In this study, we identified lncRNAs related to genome instability. By using clinical information from patients, we constructed a genome-instability-related lncRNA prognostic signature (GIRlncPSig), which can effectively predict the prognosis of colon cancer patients and contribute to the discovery of new colon cancer biomarkers.

## 2. Methods

### 2.1. Data Collection

The clinical features, somatic mutations, RNA-sequencing, and LncRNA expression of colon cancer patients were searched for and downloaded from TCGA on February 1, 2021. A total of 473 colon cancer tissues with paired mRNA and lncRNA expression profiles, somatic mutation information, and clinical information (Suppl.  Table 1) were included.

### 2.2. Set-Up of the Genome-Instability-Related lncRNA Prognostic Signature

**Figure 1:**
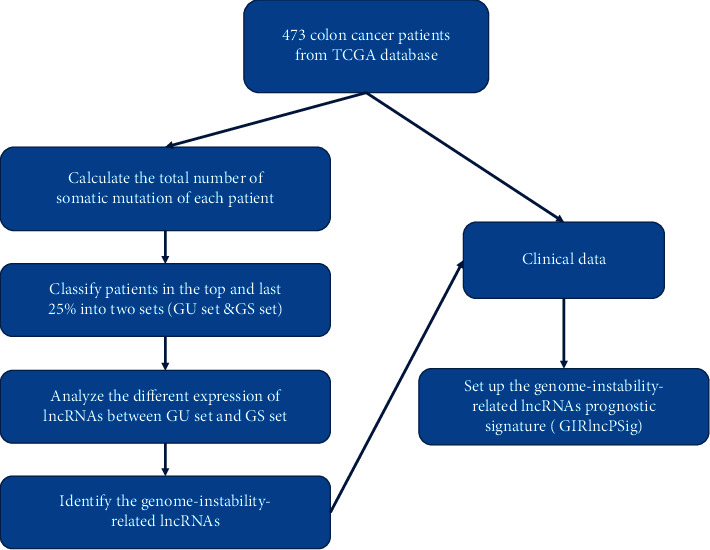
The workflow of setting up the genome-instability-related lncRNA prognostic signature (GIRlncPSig).

Using the entire transcriptome data of the patients from the TCGA database, we performed a mutator hypothesis-derived computational workflow [16] to combine lncRNA expression profiles with somatic mutation profiles. First, the total number of somatic mutations for each colon cancer patient was counted, which was then used to arrange all 473 colon cancer patients in a descending order. Based on their ranking, the top and bottom 25% of patients were defined as genome unstable (GU) and genome stable (GS) sets, respectively. The differentially expressed lncRNAs, identified as genome-instability-related lncRNAs, between the GU and GS sets were selected according to both FDR < 0.05 and |logFC| > 1 (R package “BiocManager” &“ limma”). We combined these genome-instability-related lncRNAs with the clinical data of the colon cancer patients to build a genome-instability-related lncRNA prognostic signature, i.e., GIRlncPSig (Figure 1).

### 2.3. Statistical Analysis

The hclust function (R package “limma” and “sparcl”) was used to execute hierarchical cluster analyses. Univariate and multivariate Cox analysis were applied to analyze the relationship between genome-instability-related lncRNA expression and patient overall survival (OS). Then, we constructed the GIRlncPSig: the risk score of patient = *β*gene1 × exp (gene1) + *β*gene2 × exp (gene2) + *β*gene3 × exp (gene3) + *β*gene4 × exp (gene4). In the equation, *β* represents the prognostic correlation coefficient estimated by multivariate Cox regression, while exp represents the expression value of lncRNA from the GIRlncPSig.

All colon cancer patients were randomly divided into the training or testing groups. We used the median risk score of the training group as a cutoff value to classify patients into high-risk or low-risk group. The Kaplan–Meier (K-M) method was used to draw survival curves which were tested using log-rank tests. The independence of GIRlncPSig was analyzed using multivariate Cox analysis. The time-dependent receiver operating characteristic (ROC) curve was drawn to evaluate the GIRlncPSig's performance. All of the analyses were carried out using *R* (v3.6.0).

### 2.4. Gene Function Enrichment Analysis

The correlation between the paired expression of lncRNAs and mRNAs was evaluated using Pearson correlation coefficients (R packages “BiocManager,”“limma,” and “igraph”), and the top 10 mRNAs that were coexpressed with lncRNAs were identified. The KEGG and GO functional enrichment were used to analyze the mRNAs coexpressing with the lncRNAs to predict the potential functions of the genome-instability-related lncRNAs. The KEGG and GO analyses were conducted using clusterProfiler software and the *R* (v.3.6.0) packages “colorspace,”“stringi,” and “ggplot2.”

## 3. Result

### 3.1. Identification of Genome-Instability-Related lncRNAs in Colon Cancer Patients

**Figure 2:**
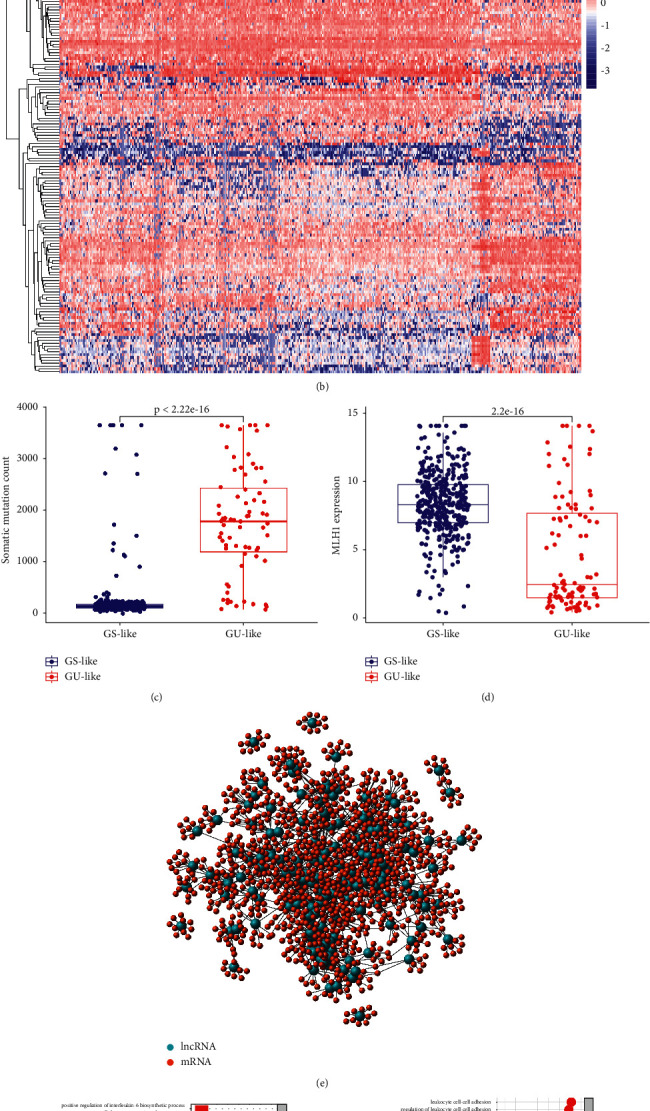
Identification of lncRNAs associated with genome instability and their functional analysis in colon cancer. (a) The differential expression of the 20 most significantly upregulated and downregulated lncRNAs in GU and GS sets of colon cancer patients. (b) Based on the expression profile of 153 genome-instability-related lncRNAs, an unsupervised clustering analysis of 473 colon cancer patients was carried out. The red cluster represents GU-like group, while the blue cluster represents the GS-like group. (c) Boxplots indicating that GU-like group have a higher number of total somatic mutations. (d) The MLH1 expression of the GU-like group was significantly lower. (e) Coexpression network of mRNAs associated with genome-instability-related lncRNAs based on the Pearson correlation coefficient analysis. The red dots are mRNAs, and the blue nodes are lncRNAs. (f) GO and (g) KEGG of mRNAs coexpressed with genome-instability-related lncRNAs.

To identify genome-instability-related lncRNAs, the total somatic mutations in each patient were calculated. Based on this quantity, in decreasing order, the top 25% of patients (*n* = 110) were classified as GU set, while the bottom 25% (*n* = 101) were classified as GS set. Then, to identify the significantly different genes, we carried out a differential expression analysis between the lncRNA expression profiles of patients in the GU and GS sets. Using the Wilcoxon test, 153 lncRNAs were identified as significantly different genes. Among them, 68 lncRNAs were upregulated and 85 were downregulated (Suppl.  Table 2).  Figure 2(a) is a heat map showing the expression of the 20 most significant upregulated and downregulated lncRNAs.

Based on the 153 differentially expressed lncRNAs, we divided the 473 patient samples from the TCGA into two groups using unsupervised hierarchical clustering analysis (Figure 2(b)). The group with higher total somatic mutations was categorized as GU-like group, while the group with lower total somatic mutations was designated as GS-like group. We compared the median total somatic mutations between these two groups and found that the GU-like group had significantly more somatic mutations compared with the GS-like group (*p* < 0.001, Figure 2(c)).Then we compared MLH1 expression (mismatch-repair-deficient colon tumors show a loss of MLH1) [17] between two groups. We found that MLH1 expression in the GU-like group was significantly lower compared with the GS-like group (*p* < 0.001; Figure 2(d)).

To determine if the 153 lncRNAs are related to genome instability, we first analyzed the correlation between them and mRNAs. Afterwards, to predict the potential functions of these lncRNAs, we applied a functional enrichment analysis of the top 10 mRNAs that were most closely correlated with each lncRNA. Next, we built a coexpression network of the lncRNAs and mRNAs, with the nodes representing the lncRNAs and mRNAs and the links indicating their correlation (Figure 2(e)). The GO analysis revealed that many of the mRNAs from the network are associated with the genome instability, including leukocyte cell-cell adhesion and DNA-binding transcription activator activity (Figure 2(f)). The KEGG pathway analysis indicated that many of enriched pathways are linked to genome instability, for example, the top three enrichment pathways of herpes simplex virus 1 infection, Th17 cell differentiation, Th1 and Th2 cell differentiation in colon cancer [18–21] (Figure 2(g)). These results imply that the 153 differentially expressed lncRNAs are potentially functionally related to genome instability and probably mediate their effects by altering the normal gene damage repair pathways via changing the balance of the lncRNA coexpressed mRNAs network. Overall, we can consider these 153 lncRNAs as genome-instability-related lncRNAs.

### 3.2. Identification of the GIRlncPSig in the Training Set

To derive the prognostic values of the genome-instability-related lncRNAs, we randomly divided the 473 colon cancer patients from the TCGA database into the training (224 patients) and the testing (249 patients) sets. More specifically, we combined the expression of the 153 genome-instability-related lncRNAs with the OS of the 224 patients in the training set using univariate Cox proportional hazard regression analysis.

**Figure 3:**
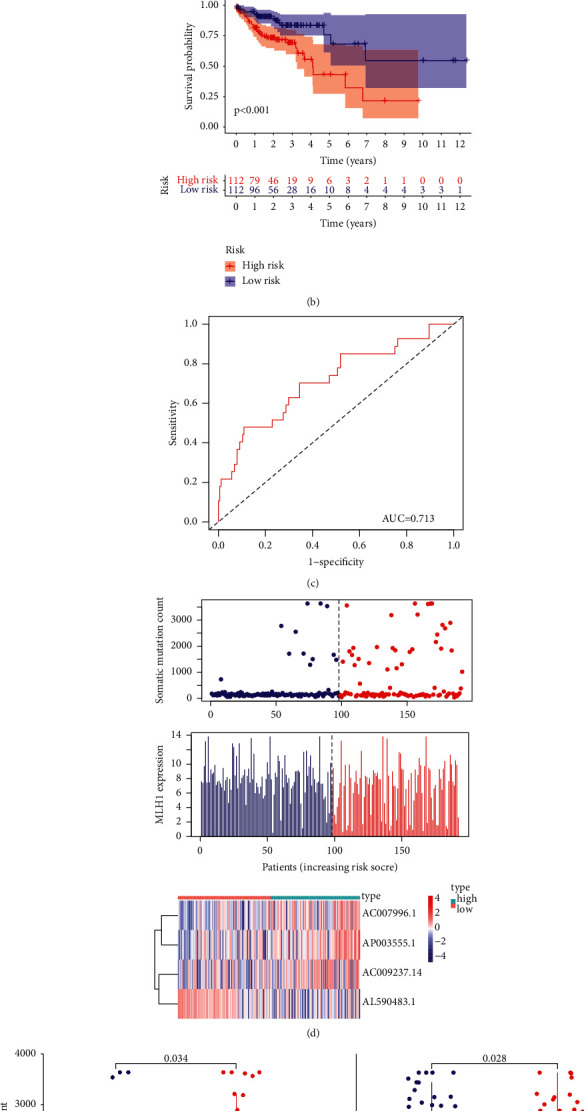
Identification of the genome-instability-related lncRNA prognostic signature (GIRlncPSig) in the training set. (a) Eight lncRNAs were found to have a close relationship with OS of colon cancer patients with univariate Cox analysis. (b) Kaplan–Meier curves showing the OS of patients in the training set. (c) Time-dependent ROC curves of GIRlncPSig at three years. (d) The distribution of somatic mutations, MLH1 expression, and lncRNA expression with increasing risk score. (e) Total somatic mutations and (f) the MLH1 expression in the high- and low-risk groups in the training set. Validation of the GIRlncPSig in the testing set.

Eight lncRNAs had a significant relationship with the prognosis of colon cancer patients (*p* < 0.05; Suppl.  Table 3; Figure 3(a)). These eight lncRNAs were used to identify those with independent prognostic values by Multivariate Cox analysis. Four of eight lncRNAs (AC007996.1, AC009237.14, AP003555.1, and AL590483.1) were recognized as independent prognostic lncRNAs (Table 1). We built the GIRlncPSig to evaluate the prognosis risk of colon cancer patients using the coefficients from the multivariate Cox analysis. Accordingly, the risky score of the GIRlncPSig = (0.314209 × exp AC007996.1) + (0.261624 × exp AC009237.14) + (0.504761 × exp AP003555.1) + (−1.01884 × exp AL590483.1). Note that the coefficients of AC007996.1, AC009237.14, and AP003555.1 were positive, implying that these genes may be prognosis risky factors because the higher their expression, the higher risk scores and poorer prognosis. Conversely, the AL590483.1 coefficient is negative, so it may be a protective factor in that the higher its expression, the lower risk score and longer the patients' survival.

Using the GIRlncPSig score, we calculated all patients in the training set. All patients were classified into high-risk and low-risk groups with median GIRlncPSig risk score. From the K-M analysis, patients in the low-risk group had better long-term prognosis than patients in the high-risk group (*p*=<0.001; Figure 3(b)). The area under curve (AUC) of time dependent ROC curve was 0.713 at three years (Figure 3(c)). We also analyzed the levels of somatic mutations and MLH1 expression in two groups (Figure 3(d)). Patients in the high-risk group had significantly more somatic mutations than the low-risk group (*p*=0.034, Figure 3(e)). Furthermore, the high-risk group had a lower level of MLH1 expression significantly (*p*=0.028; Figure 3(f)).

To validate the GIRlncPSig, the risk score of each patient in the testing set was calculated. The median risk score was set as the cutoff value in classifying patients from the testing set into the high-risk group and low-risk groups.

**Figure 4:**
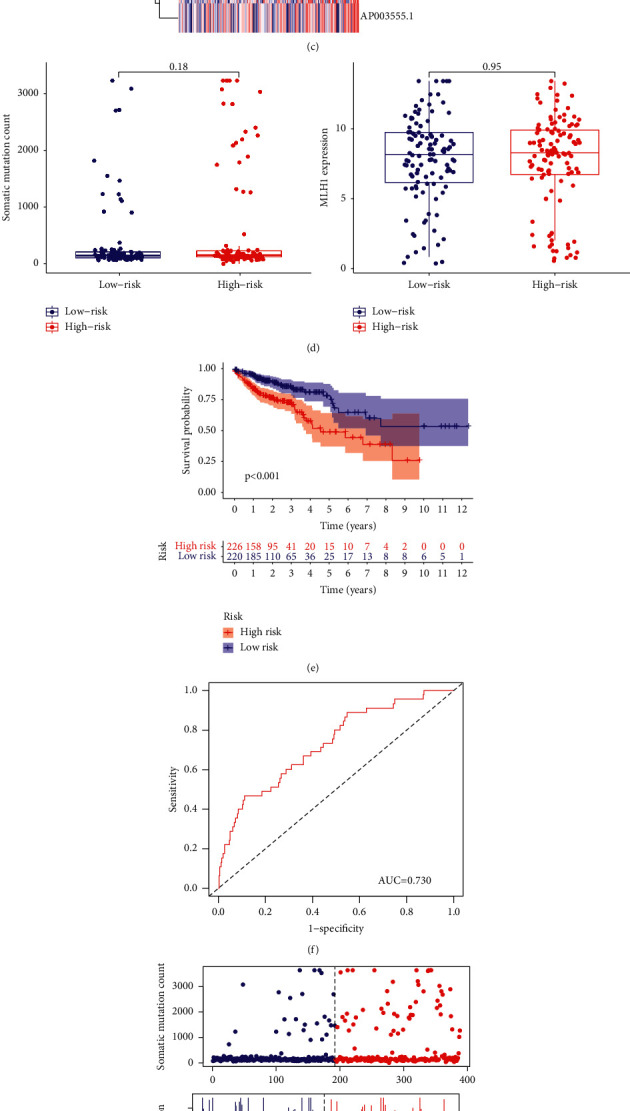
Evaluation of the GIRlncPSig in the testing and TCGA sets. (a) K-M curves indicating OS in high- and low-risk group patients as classified using the GIRlncPSig in the testing and (e) TCGA sets. (b) Time-dependent ROC curves analysis of the GIRlncPSig at three years in the testing and (f) TCGA sets. (c) The distribution of somatic mutation, MLH1 expression, and lncRNA expression with increasing risk scores in the testing and (g) TCGA sets. (d) Total somatic mutation and MLH1 expression levels in the high- and low-risk groups in the testing and (h) TCGA sets.

Low-risk group patents had better long-term prognosis (*p*=0.031; Figure 4(a)). The AUC of time-dependent ROC curve was 0.746 at three years (Figure 4(b)). Next, we compared somatic mutations and MLH1 expression levels in the two groups (Figure 4(c)). The number of somatic mutations was not significantly different in the two risk groups (*p*=0.18; Figure 4(d)). However, MLH1 expression was also not significantly different (*p*=0.95; Figure 4(d)).

Similar to before, based on the median risk value, all the patients in the TCGA set were also divided into the high-risk group and low-risk group. Accordingly, the OS of low-risk group was significantly higher (*p* < 0.001; Figure 4(e)). The AUC of time dependent ROC curves of the TCGA set was 0.730 at 3 years (Figure 4(f)). The number of somatic mutations and the expression level of MLH1 in the two risk groups are illustrated in Figure 4(g). The low-risk group patients had significantly more somatic mutations compared with the high-risk group patients (*p*=0.015; Figure 4(h)). However, MLH1 expression was marginally lower in the high-risk group patients (*p*=0.18; Figure 4(h)).

### 3.3. Comparison of the GIRlncPSig with the Preexisting lncRNA Prognostic Signatures

**Figure 5:**
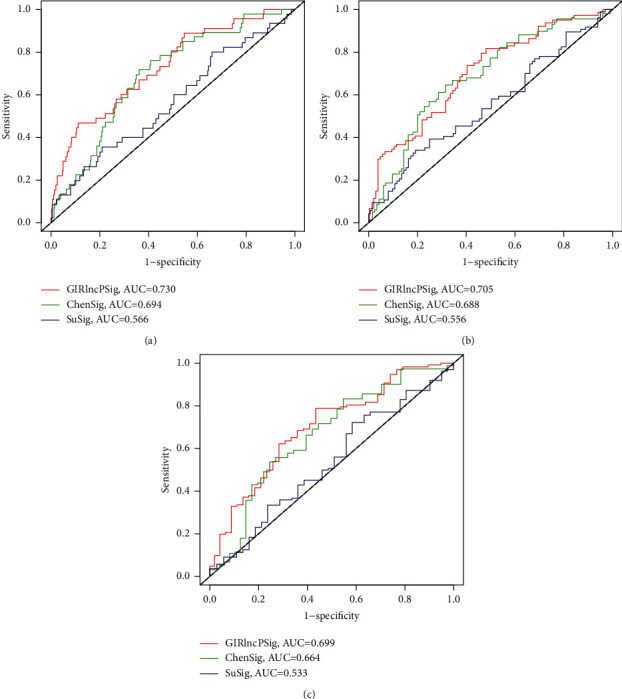
Comparison of area under the curve of time-dependent ROC curves at one, three, and five years of OS for GIRlncPSig, SuSig, and ChenSig.

To further validate the GIRlncPSig, we compared its prediction performance with two recently published lncRNA prognostic signatures: the recurrence-associated six-lncRNA prognostic signature from Su (referred to as SuSig) [22] and autophagy-related 10-lncRNA prognostic signature from Chen (referred to as ChenSig) [23]. The AUC of the time-dependent ROC curve at one, three, five years of OS for the GIRlncPSig is 0.730, 7.05, and 0.699 which is significantly higher than those of SuSig (AUC = 0.566, 0.556, 0.533) and ChenSig (AUC = 0.694, 0.688, 0.664) (Figures 5(a)–5(c)). Furthermore, the number of lncRNAs of our GIRlncPSig is less than that of Su's (six lncRNAs) and Chen's (ten lncRNAs) prognostic signatures. These results demonstrate that the GIRlncPSig has a better prognostic performance than two other recently published lncRNA prognostic signatures.

### 3.4. Independence of the GIRlncPSig of Other Clinical Factors

To assess if the GIRlncPSig was independent of common clinical variables, we analyzed age, gender, pathologic stage, and GIRlncPSig using univariate Cox regression analysis. The results suggest that GIRlncPSig, pathologic stage, and age were significantly related to OS. A multivariate Cox regression analysis was used to analyze GIRlncPSig, pathologic stage, and age—factors that were significantly correlated to OS in the univariate Cox analysis. Here, the results indicated that the GIRlncPSig was also significantly related with OS (Table 2).

**Figure 6:**
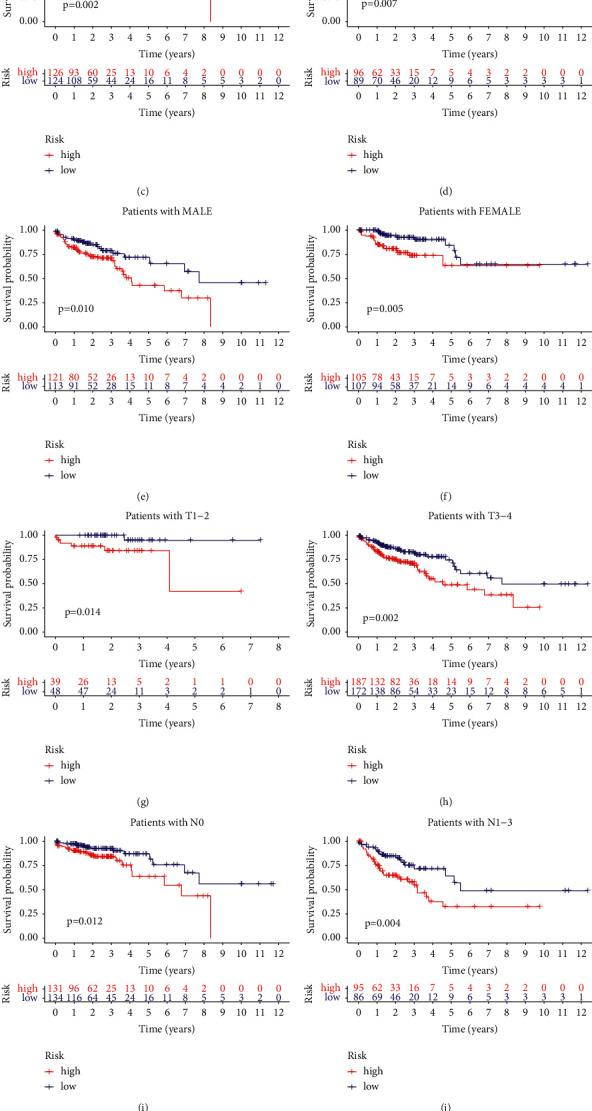
Stratification analyses by age, pathologic stage, gender, and GIRlncPSig. (a) K-M curves of high- and low-risk for young and (b) old group patients. (c) K-M curves of high- and low-risk for early-stage and (d) late-stage group patients. (e) K-M curves of high- and low-risk for male and (f) female group patients. (g) K-M curves of high- and low-risk for early-stage and (h) late-stage group patients. (i) K-M curves of high- and low-risk for N0 stage and (j) N1-3 stage group patients. (k) K-M curves of high- and low-risk for M0 stage and (l) M1 stage group patients.

Next, we used stratification analysis to evaluate whether the GIRlncPSig had a prognostic value that was independent of the pathologic stage, age, and gender. All colon cancer patients in the TCGA set were stratified into either a young patient group or an old patient group using the cutoff age of 65 years. Based on the median GIRlncPSig score, the patients were further divided into high-risk or low-risk groups. Both young (*p*=0.009; Figure 6(a)) and old (*p*=0.003; Figure 6(b)) patients of the two risk groups had different OS.

All colon cancer patients were stratified using pathologic stage. We considered pathologic stage I or II patients as the early-stage group, while patients at pathologic stage III or IV were designated as the late-stage group. We classified each patient in the early-stage and late-stage groups as high-risk or low-risk groups based on the median GIRlncPSig score. OS was significantly different in both risk groups in early-stage (*p*=0.002; Figure 6(c)) and late-stage (*p*=0.007; Figure 6(d)) group patients.

Finally, we stratified the patients using gender (male/female) (Figures 6(e) and 6(f)), T (T1–2/T3–4) (Figures 6(g) and 6(h)), N (N0/N1–3) (Figures 6(i) and 6(j)), and M (M0/M1) (Figures 6(k) and 6(l)). We also classified the patients into high-risk and low-risk groups using the median GIRlncPSig score. Compared with the high-risk group in each of the stratifications, the low-risk group had better OS (*p* < 0.05) (Suppl.  Table 1). Therefore, the GIRlncPSig is an independent prognostic factor in colon cancer patients.

### 3.5. The GIRlncPSig Is Related to BRAF and DPYD Mutation Status

**Figure 7:**
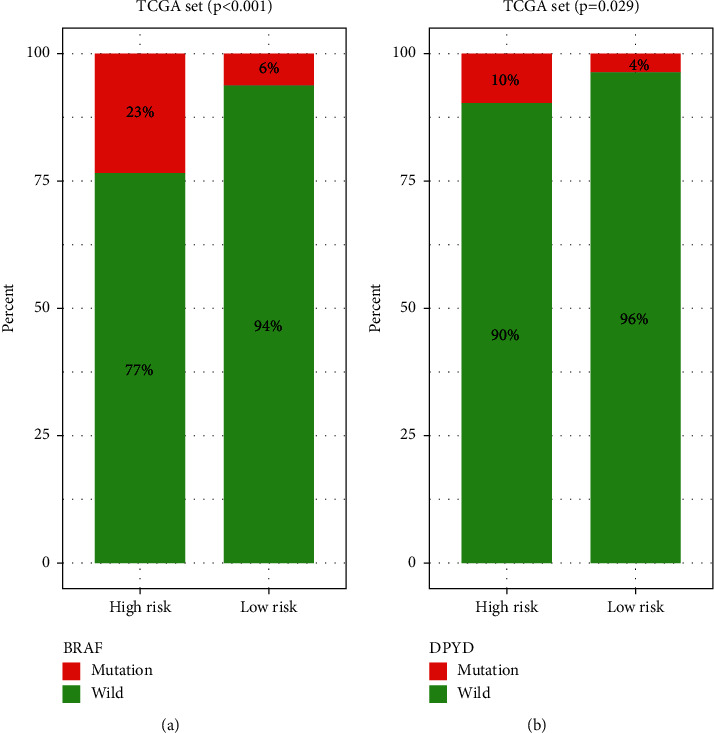
Correlation of GIRlncPSig with (a) BRAF and (b) DPYD somatic mutation status.

To evaluate whether the GIRlncPSig is related to some common mutations, we analyzed the relationship between the GIRlncPSig and some common mutations frequently measured in the clinic. The results revealed that the mutation rates of the BRAF and DPYD genes were significantly higher in the high-risk group in the TCGA set (*p* < 0.05; Figures 7(a) and 7(b)). BRAF and DPYD mutations can be used as markers to guide treatment in colon cancer. These results indicate that the GIRlncPSig is related to BRAF and DPYD gene mutation status. Hance, the GIRlncPSig may be used as a mutation marker for the BRAF and DPYD gene.

## 4. Discussion

With the rapid development of immunotherapy, the treatment of colon cancer is not limited to traditional surgery, chemotherapy, and radiotherapy. Increasing numbers of patients are beginning to receive immunotherapy. Immunotherapy is closely related to genome instability, a common feature of most cancers [24, 25]. In 2017, the FDA approved immunotherapy for the treatment of colorectal cancer patients that are mismatch-repair-deficient or have high levels of microsatellite instability. LncRNA has been demonstrated to affect the biological behavior of tumors. Their dysregulation may be related to the tumors progression, and they may also have a prognostic predictive effect of cancer patients [26, 27]. Much of the current data indicates that lncRNAs are associated with tumor genome instability [28, 29]. Although some progress has been made, the relationship between lncRNAs and genome instability in colon cancer as well as their clinical significance remains to be further explored.

In this study, we identified 153 genome-instability-related lncRNAs by analyzing the differential expression of lncRNAs between patients in the top or bottom 25% of total somatic mutations. We then functionally analyzed mRNAs coexpressed with those 153 genome-instability-related lncRNAs, with the results indicating that these coexpressed mRNAs were enriched in leukocyte cell-cell adhesion and DNA-binding transcription activator activity, which are associated with genome instability [30, 31].We also found the enrichment of coexpressed mRNAs involved in cytokine−cytokine receptor interaction, Th1 and Th2 cell differentiation, PD−L1 expression, and PD−1 checkpoint pathway in cancer, which are also associated with cancer immunotherapy and genome instability [32–35]. Then we tested if the genome-instability-related lncRNAs can predict clinical outcome. Using univariate and multivariate Cox analyses, we built GIRlncPSig, which is composed of four lncRNAs (AC007996.1, AC009237.14, AP003555.1, and AL590483.1). However, their biological functions have yet to be reported. We used the GIRlncPSig to divide patients from a training or testing set into high- or low-risk group; in either set, survival was significantly different in the two groups. Additionally, GIRlncPSig was related to total somatic mutations and MLH1 expression in colon cancer patients. The Cox analyses results involving pathologic stage age, gender, and GIRlncPSig indicated that GIRlncPSig was independent of common clinical variables. Similarly, the stratification analysis based on age, pathologic stage, or gender also demonstrated that GIRlncPSig was an independent prognostic factor. Finally we analyzed the relationship between GIRlncPSig and some common mutations frequently clinically measured. We found that BRAF and DPYD mutation rates in the high-risk group were significantly higher, indicating that the GIRlncPSig may help predict the BRAF and DPYD mutation in colon cancer patients. Seligmann et al. [36] reported that the OS of BRAF-mutant-type colorectal cancer patients was shorter than wild type, suggesting that BRAF mutations may be a prognostic factor for colorectal cancer patients. Hideo et al. [37] observed that DPYD expression and activity in tumor cells in vivo are related to the antitumor sensitivity of fluorouracil; thus, high DPYD expression may lead to fluorouracil drug resistance. Altogether, the GIRlncPSig may be used as a mutation marker for the BRAF and DPYD genes and may be beneficial clinically.

Although our study rigorously analyzed the prognostic value of genome-instability-related lncRNAs in colon cancer, it still has some limitations. The GIRlncPSig was verified in the TCGA database, but we were unable to find any of these four lncRNAs on other platforms as the Gene Expression Omnibus, so more independent datasets are needed to verify the robustness and repeatability of the GIRlncPSig. In addition, GIRlncPSig was identified based on somatic mutation counts of each patient; accordingly, further studies are necessary to identify the specific mechanisms of the GIRlncPSig in affecting genome instability.

## 5. Conclusion

In this work, we combined somatic mutation profiles with lncRNA expression profiles in colon cancer patients from the TCGA database and identified 153 genome-instability-related lncRNAs. Then, we combined the genome-instability-related lncRNAs with the prognosis of colon cancer patients to build the GIRlncPSig. Altogether, the GIRlncPSig may improve prognosis prediction, mark genome instability, and have clinical benefits for colon cancer patients.

## Figures and Tables

**Table 1:** The genome-instability-related lncRNAs of prognostic signature.

LncRNA	Coef	HR	95% CI	*p* value

AC007996.1	0.314209	1.369176	0.952473–1.968185	0.0897
AC009237.14	0.261624	1.299038	1.152733–1.463911	1.78E-05
AP003555.1	0.504761	1.65659	1.275865–2.150925	0.000152
AL590483.1	−1.01884	0.361013	0.164662–0.791503	0.010967

HR: hazard ratio, CI: confidence interval, and coef: coefficient value.

**Table 2:** Cox analysis of the GIRlncPSig and normal clinical factors with OS in TCGA database.

Variables	Univariable model			Multivariable model		
	HR	95% CI	*p*-value	HR	95% CI	*p*-value

GILncSig	1.271	1.188–1.359	<0.001	1.023	1.002 1.044	0.033
Age	1.044	1.016–1.073	0.0016	1.037	1.018 1.057	<0.001
Gender	1.049	0.597–1.844	0.865			
Pathologic stage	1.961	1.422–2.704	<0.001	2.231	1.747 2.848	<0.001

HR: hazard ratio; CI: confidence interval.

## Data Availability

Publicly available datasets were analyzed in this study (https://portal.gdc.cancer.gov/).

## Supplementary Materials

Supplementary Materials Suppl. Table 1: correlation between risk level and clinicopathologic characteristics of colon cancer patients. Suppl. Table 2: 153 genome-instability-related lncRNAs in colon cancer patients. Suppl. Table 3: the lncRNAs associated with genome instability significantly related to the prognosis of colon cancer patients analyzed by univariate Cox proportional hazard regression analysis .
