# Supplementary material for: Identification of a Four-lncRNA Prognostic Signature for Colon Cancer Based on Genome Instability
Source: J Oncol. 2021 Sep 21;2021:7408893. doi: 10.1155/2021/7408893 (PMC8478558; doi:10.1155/2021/7408893)
Supplement: Supplementary Materials — Suppl. Table 1: correlation between risk level and clinicopathologic characteristics of colon cancer patients. Suppl. Table 2: 153 genome-instability-related lncRNAs in colon cancer patients. Suppl. Table 3: the lncRNAs associated with genome instability significantly related to the prognosis of colon cancer patients analyzed by univariate Cox proportional hazard regression analysis . [file 7408893.f1.zip › 7408893.f1/Supplementary description.docx]

**Supplementary description:**

Supplementary Table 1. Correlation between risk level and clinicopathologic characteristics of colon cancer patients ^a^.

Supplementary Table 2 153 genome instability related lncRNAs in colon cancer patients

Supplementary table 3 The lncRNAs associated with genome instability significantly related to the prognosis of colon cancer patients analyzed by univariate Cox proportional hazard regression analysis
