# Supplementary material for: Identification of a Four-lncRNA Prognostic Signature for Colon Cancer Based on Genome Instability
Source: J Oncol. 2021 Sep 21;2021:7408893. doi: 10.1155/2021/7408893 (PMC8478558; doi:10.1155/2021/7408893)
Supplement: Supplementary Materials — Suppl. Table 1: correlation between risk level and clinicopathologic characteristics of colon cancer patients. Suppl. Table 2: 153 genome-instability-related lncRNAs in colon cancer patients. Suppl. Table 3: the lncRNAs associated with genome instability significantly related to the prognosis of colon cancer patients analyzed by univariate Cox proportional hazard regression analysis . [file 7408893.f1.zip › 7408893.f1/supplementary table 1 (1).docx]

Supplementary Table 1. Correlation between risk level and clinicopathologic characteristics of colon cancer patients ^a^.

| Clinicopathological characteristics | Variable | Risk level | | *p value* |
| --- | --- | --- | --- | --- |
|  |  | High | Low |  |
| No |  | 226 | 220 |  |
| Survival status | Alive | 164 (72.6 %) | 186 (84.5 %) | 0.002 |
|  | Dead | 62 (27.4 %) | 34 (15.5 %) |  |
| Age (years) | <=65 | 92 (40.7 %) | 91 (41.4 %) | 0.888 |
|  | >65 | 134 (59.3 %) | 129 (58.6 %) |  |
| Gender | Female | 109 (48.2 %) | 103 (46.8 %) | 0.765 |
|  | Male | 117 (51.8 %) | 117 (53.2 %) |  |
| Pathological stage | Stage I | 34 (15.0 %) | 41 (18.6 %) | 0.590 |
|  | Stage II | 92 (40.7 %) | 83 (37.3 %) |  |
|  | Stage III | 67 (29.6 %) | 57 (25.9 %) |  |
|  | Stage IV | 29 (12.8 %) | 32 (14.5 %) |  |
|  | Unknow | 4 (1.8 %) | 7 (3.2 %) |  |
| T | T1 | 4 (1.8 %) | 6 (2.7 %) | 0.638 |
|  | T2 | 35 (15.5 %) | 41 (18.6 %) |  |
|  | T3 | 159 (70.4 %) | 144 (65.5 %) |  |
|  | T4 | 28 (12.4 %) | 28 (12.7 %) |  |
|  | Tis | 0 (0 %) | 1 (0.5 %) |  |
| M | M0 | 166 (73.5 %) | 163 (74.1 %) | 0.692 |
|  | M1 | 29 (12.8 %) | 32 (14.5 %) |  |
|  | MX | 31 (13.7 %) | 25 (11.4 %) |  |
| N | N0 | 131 (58.0 %) | 134 (60.9 %) | 0.736 |
|  | N1 | 52 (23.0 %) | 50 (22.7 %) |  |
|  | N2 | 43 (19.0 %) | 36 (16.4 %) |  |

Abbreviations: T: tumor, N: nodes, M: metastases; a Chi-square test, * *p* < 0.05, ** *p* < 0.01.
