# Supplementary material for: Identification of a Four-lncRNA Prognostic Signature for Colon Cancer Based on Genome Instability
Source: J Oncol. 2021 Sep 21;2021:7408893. doi: 10.1155/2021/7408893 (PMC8478558; doi:10.1155/2021/7408893)
Supplement: Supplementary Materials — Suppl. Table 1: correlation between risk level and clinicopathologic characteristics of colon cancer patients. Suppl. Table 2: 153 genome-instability-related lncRNAs in colon cancer patients. Suppl. Table 3: the lncRNAs associated with genome instability significantly related to the prognosis of colon cancer patients analyzed by univariate Cox proportional hazard regression analysis . [file 7408893.f1.zip › 7408893.f1/supplementary table 2 (1).docx]

**Supplementary Table 2**  **153 genome instability related lncRNAs in colon cancer patients**

| Type | Genes | LogFC | P Value | FDR |
| --- | --- | --- | --- | --- |
| Up-regulated | LINC01871 | 1.00496 | 7.55E-07 | 6.76E-06 |
|  | AC026470.2 | 1.00524 | 0.004619 | 0.012128 |
|  | AP001107.4 | 1.011199 | 0.003097 | 0.00865 |
|  | AL162724.2 | 1.017195 | 0.012498 | 0.028175 |
|  | USP30-AS1 | 1.0215 | 2.46E-07 | 2.53E-06 |
|  | C1orf195 | 1.03914 | 0.008092 | 0.019479 |
|  | AC129492.1 | 1.048396 | 0.000425 | 0.001622 |
|  | LINC00896 | 1.050036 | 0.000994 | 0.003294 |
|  | HAR1B | 1.050062 | 1.33E-06 | 1.09E-05 |
|  | AC100791.2 | 1.051057 | 0.001761 | 0.005401 |
|  | AC020656.2 | 1.057851 | 0.014733 | 0.032606 |
|  | LINC01094 | 1.084907 | 1.04E-06 | 8.84E-06 |
|  | AC087286.2 | 1.085143 | 0.018883 | 0.039922 |
|  | AC253536.3 | 1.095924 | 0.008611 | 0.020515 |
|  | AC027514.2 | 1.098959 | 0.005008 | 0.012918 |
|  | LINC02100 | 1.10686 | 0.017737 | 0.037858 |
|  | NPTN-IT1 | 1.112369 | 0.010376 | 0.024083 |
|  | AC130456.3 | 1.126983 | 0.000575 | 0.002059 |
|  | LOXL1-AS1 | 1.129559 | 2.59E-09 | 4.94E-08 |
|  | AC026202.2 | 1.171055 | 0.003344 | 0.009165 |
|  | AL136115.2 | 1.179456 | 0.011159 | 0.025607 |
|  | HLA-DQB1-AS1 | 1.186241 | 4.92E-05 | 0.000254 |
|  | AC091588.1 | 1.196537 | 0.001307 | 0.0042 |
|  | AC115522.1 | 1.237826 | 7.09E-09 | 1.14E-07 |
|  | AC005256.1 | 1.241856 | 4.55E-05 | 0.000238 |
|  | AC007996.1 | 1.243463 | 8.70E-13 | 4.55E-11 |
|  | AGAP1-IT1 | 1.270354 | 6.82E-08 | 8.37E-07 |
|  | AC012317.1 | 1.27224 | 7.48E-05 | 0.000367 |
|  | AC005911.1 | 1.279498 | 3.74E-11 | 1.19E-09 |
|  | AC007991.2 | 1.288943 | 0.000633 | 0.002245 |
|  | AC090181.2 | 1.316465 | 1.08E-08 | 1.62E-07 |
|  | MIAT | 1.317381 | 3.16E-06 | 2.35E-05 |
|  | AC025423.1 | 1.339243 | 0.002653 | 0.00758 |
|  | AP003555.1 | 1.342529 | 7.34E-05 | 0.000361 |
|  | AC112484.3 | 1.35306 | 0.000315 | 0.00127 |
|  | AC009163.6 | 1.364131 | 0.000159 | 0.000697 |
|  | AP005899.1 | 1.387608 | 1.31E-06 | 1.08E-05 |
|  | AL606834.1 | 1.390296 | 1.98E-09 | 3.86E-08 |
|  | AC023825.2 | 1.405808 | 4.20E-05 | 0.000224 |
|  | LINC02041 | 1.425002 | 2.71E-09 | 4.99E-08 |
|  | LIMS1-AS1 | 1.457319 | 0.011263 | 0.025789 |
|  | AC004771.4 | 1.457833 | 0.003674 | 0.009886 |
|  | LINC00941 | 1.498321 | 1.54E-07 | 1.67E-06 |
|  | AP000753.2 | 1.500584 | 0.000126 | 0.00057 |
|  | AC005392.2 | 1.503323 | 0.000346 | 0.00137 |
|  | LINC01443 | 1.628721 | 0.000338 | 0.001352 |
|  | AC064801.1 | 1.645743 | 2.05E-08 | 2.89E-07 |
|  | XXYLT1-AS2 | 1.688819 | 6.79E-09 | 1.10E-07 |
|  | AL121761.1 | 1.688903 | 6.81E-05 | 0.000337 |
|  | LINC02489 | 1.730159 | 5.00E-07 | 4.71E-06 |
|  | AL157871.2 | 1.748065 | 2.73E-07 | 2.75E-06 |
|  | AL139022.1 | 1.75853 | 0.00156 | 0.004908 |
|  | UNC5B-AS1 | 1.770392 | 5.00E-05 | 0.000257 |
|  | AC114760.2 | 1.772652 | 0.000344 | 0.00137 |
|  | LINC02195 | 1.796004 | 5.65E-14 | 4.16E-12 |
|  | LUCAT1 | 1.832257 | 0.008426 | 0.020103 |
|  | AC245128.3 | 1.918442 | 0.001145 | 0.003734 |
|  | AL138789.1 | 2.000267 | 0.000524 | 0.00193 |
|  | AL022316.1 | 2.063932 | 2.17E-09 | 4.18E-08 |
|  | TFAP2A-AS1 | 2.080816 | 1.60E-16 | 4.31E-14 |
|  | AC010998.3 | 2.120278 | 0.000112 | 0.000525 |
|  | AC036176.3 | 2.292384 | 0.001615 | 0.005049 |
|  | AFAP1-AS1 | 2.350591 | 0.001978 | 0.005957 |
|  | AC022784.1 | 2.407417 | 1.85E-10 | 4.83E-09 |
|  | LINC02446 | 2.827584 | 2.75E-15 | 3.70E-13 |
|  | AC092112.1 | 3.246627 | 2.82E-05 | 0.000159 |
|  | AC008514.1 | 3.623936 | 6.08E-08 | 7.59E-07 |
|  | DLGAP1-AS5 | 5.407678 | 2.36E-06 | 1.84E-05 |
| Down-regulated | FENDRR | -1.00006 | 6.58E-13 | 3.55E-11 |
|  | AC105219.1 | -1.0098 | 8.60E-05 | 0.000416 |
|  | LINC01006 | -1.01268 | 5.68E-15 | 6.46E-13 |
|  | AC020663.2 | -1.01318 | 7.59E-09 | 1.19E-07 |
|  | AC022613.1 | -1.02652 | 1.24E-09 | 2.61E-08 |
|  | AL161772.1 | -1.03071 | 1.80E-10 | 4.78E-09 |
|  | LINC01558 | -1.03395 | 7.13E-08 | 8.62E-07 |
|  | ZNF529-AS1 | -1.03481 | 4.19E-10 | 1.01E-08 |
|  | AL133520.1 | -1.03832 | 4.12E-12 | 1.76E-10 |
|  | LINC01003 | -1.03858 | 2.97E-15 | 3.70E-13 |
|  | BOLA3-AS1 | -1.04018 | 1.83E-05 | 0.000112 |
|  | AC009061.1 | -1.05235 | 0.000154 | 0.000681 |
|  | AC064807.2 | -1.05558 | 3.82E-07 | 3.66E-06 |
|  | AC116345.1 | -1.05991 | 0.000801 | 0.002713 |
|  | AC008750.4 | -1.06123 | 0.014758 | 0.032617 |
|  | HAS2-AS1 | -1.06236 | 5.50E-09 | 9.27E-08 |
|  | LINC02563 | -1.07069 | 4.76E-08 | 6.17E-07 |
|  | AL590483.1 | -1.07373 | 6.72E-07 | 6.18E-06 |
|  | AL391056.1 | -1.07374 | 8.80E-09 | 1.34E-07 |
|  | CASC19 | -1.08144 | 5.26E-05 | 0.00027 |
|  | DIO3OS | -1.1009 | 3.67E-11 | 1.19E-09 |
|  | LINC01315 | -1.11693 | 9.79E-11 | 2.73E-09 |
|  | AC020659.1 | -1.13472 | 3.47E-06 | 2.55E-05 |
|  | AC124067.4 | -1.14866 | 1.72E-13 | 1.12E-11 |
|  | AC009237.15 | -1.15673 | 3.23E-07 | 3.19E-06 |
|  | AL355312.3 | -1.16742 | 5.53E-06 | 3.93E-05 |
|  | AC090579.1 | -1.16817 | 2.75E-11 | 9.33E-10 |
|  | ZNF503-AS1 | -1.17366 | 4.91E-08 | 6.32E-07 |
|  | DPP10-AS1 | -1.1833 | 0.000147 | 0.000652 |
|  | AL035661.1 | -1.18541 | 3.50E-13 | 1.95E-11 |
|  | AC123023.1 | -1.18547 | 5.77E-12 | 2.40E-10 |
|  | LINC00543 | -1.19033 | 3.45E-16 | 6.22E-14 |
|  | SMIM2-AS1 | -1.20652 | 4.55E-09 | 7.83E-08 |
|  | AC106876.1 | -1.20654 | 6.74E-16 | 1.09E-13 |
|  | AC109446.3 | -1.21098 | 7.34E-11 | 2.09E-09 |
|  | AL139384.1 | -1.22133 | 6.00E-10 | 1.41E-08 |
|  | AL022313.2 | -1.23091 | 8.22E-07 | 7.20E-06 |
|  | AC080129.2 | -1.24906 | 3.73E-10 | 9.29E-09 |
|  | AP005271.1 | -1.26458 | 3.25E-08 | 4.28E-07 |
|  | AC009237.14 | -1.27444 | 6.54E-12 | 2.58E-10 |
|  | LINC02418 | -1.29813 | 2.61E-10 | 6.59E-09 |
|  | AL133370.1 | -1.3042 | 1.18E-06 | 9.77E-06 |
|  | LINC02487 | -1.31436 | 8.15E-14 | 5.74E-12 |
|  | AC254629.1 | -1.32406 | 6.21E-15 | 6.46E-13 |
|  | AL121829.2 | -1.32847 | 2.38E-06 | 1.85E-05 |
|  | HNF4A-AS1 | -1.35267 | 0.000693 | 0.002419 |
|  | RHPN1-AS1 | -1.35677 | 5.96E-11 | 1.72E-09 |
|  | AP004608.1 | -1.36595 | 6.23E-12 | 2.52E-10 |
|  | AL121895.2 | -1.37493 | 1.55E-09 | 3.10E-08 |
|  | AL121832.1 | -1.37497 | 1.61E-08 | 2.33E-07 |
|  | LINC01807 | -1.38678 | 3.34E-05 | 0.000184 |
|  | AC026801.2 | -1.38783 | 2.85E-13 | 1.71E-11 |
|  | AC017074.1 | -1.39724 | 4.24E-17 | 2.63E-14 |
|  | SATB2-AS1 | -1.39862 | 1.02E-13 | 6.87E-12 |
|  | RARA-AS1 | -1.40655 | 1.59E-06 | 1.28E-05 |
|  | LHFPL3-AS2 | -1.42691 | 6.88E-06 | 4.74E-05 |
|  | OSER1-DT | -1.42792 | 7.99E-20 | 1.29E-16 |
|  | AC055717.2 | -1.44103 | 5.18E-11 | 1.55E-09 |
|  | AC009812.1 | -1.44366 | 2.64E-14 | 2.38E-12 |
|  | AL390198.1 | -1.4531 | 2.02E-07 | 2.14E-06 |
|  | AL162582.1 | -1.46285 | 2.60E-07 | 2.66E-06 |
|  | AL365226.1 | -1.5285 | 2.80E-06 | 2.12E-05 |
|  | AP000785.1 | -1.56848 | 2.02E-07 | 2.14E-06 |
|  | LINC02441 | -1.58958 | 6.38E-15 | 6.46E-13 |
|  | PTPRD-AS1 | -1.61725 | 3.49E-14 | 2.93E-12 |
|  | AC108134.3 | -1.61955 | 1.21E-11 | 4.44E-10 |
|  | BOK-AS1 | -1.63202 | 0.008031 | 0.019361 |
|  | LINC01082 | -1.64335 | 4.01E-14 | 3.09E-12 |
|  | AL390208.1 | -1.65651 | 0.002157 | 0.006425 |
|  | AL117382.2 | -1.66199 | 1.37E-16 | 4.31E-14 |
|  | LINC00525 | -1.73714 | 2.25E-15 | 3.32E-13 |
|  | AL117382.1 | -1.78744 | 1.68E-12 | 7.78E-11 |
|  | AC093585.1 | -1.8136 | 1.17E-10 | 3.22E-09 |
|  | TUSC8 | -1.84786 | 4.87E-17 | 2.63E-14 |
|  | LINC01811 | -1.91598 | 3.04E-09 | 5.54E-08 |
|  | LINC00654 | -2.01516 | 1.88E-13 | 1.17E-11 |
|  | AC114296.1 | -2.09399 | 1.18E-12 | 5.83E-11 |
|  | AC010280.1 | -2.10658 | 2.48E-10 | 6.37E-09 |
|  | AP003774.2 | -2.333 | 1.06E-16 | 4.31E-14 |
|  | AC136475.9 | -2.50997 | 2.98E-07 | 2.96E-06 |
|  | AC078993.1 | -2.59355 | 1.73E-11 | 6.08E-10 |
|  | AC026336.3 | -2.83814 | 3.60E-11 | 1.19E-09 |
|  | AC007608.2 | -3.01637 | 0.003123 | 0.008707 |
|  | AC108865.2 | -3.79013 | 5.13E-06 | 3.67E-05 |
|  | AC108865.1 | -4.04063 | 2.63E-09 | 4.95E-08 |

Abbreviations: FC Fold Change, FDR false discovery rate
