# Supplementary material for: Identification of a Four-lncRNA Prognostic Signature for Colon Cancer Based on Genome Instability
Source: J Oncol. 2021 Sep 21;2021:7408893. doi: 10.1155/2021/7408893 (PMC8478558; doi:10.1155/2021/7408893)
Supplement: Supplementary Materials — Suppl. Table 1: correlation between risk level and clinicopathologic characteristics of colon cancer patients. Suppl. Table 2: 153 genome-instability-related lncRNAs in colon cancer patients. Suppl. Table 3: the lncRNAs associated with genome instability significantly related to the prognosis of colon cancer patients analyzed by univariate Cox proportional hazard regression analysis . [file 7408893.f1.zip › 7408893.f1/supplementary table 3 (1).docx]

**Supplementary table 3 The lncRNAs associated with genome instability significantly related to the prognosis of colon cancer patients analyzed by univariate Cox proportional hazard regression analysis**

| id | HR | 95% CI | P-value |
| --- | --- | --- | --- |
| AC007996.1 | 1.481728 | 1.077516-2.037573 | 0.01555 |
| LINC00941 | 1.288858 | 1.086181-1.529353 | 0.003649 |
| UNC5B-AS1 | 1.058582 | 1.000445-1.120096 | 0.048221 |
| `LINC02041 | 1.214832 | 1.036328-1.424081 | 0.016393 |
| AC009237.14 | 1.157548 | 1.036111-1.293218 | 0.009673 |
| BOLA3-AS1 | 1.360038 | 1.016937-1.818896 | 0.038154 |
| AP003555.1 | 1.643774 | 1.301248-2.076464 | 3.06E-05 |
| AL590483.1 | 0.450373 | 0.22849-0.887722 | 0.021225 |

Abbreviations: HR Hazard Ratio, CI Confidence Interval
